# Epidermoid cyst in an intrapancreatic accessory spleen with abnormally high CEA level in cyst fluid: a case report

**DOI:** 10.4322/acr.2021.369

**Published:** 2022-04-14

**Authors:** Chun-hai Lo, Po-man Tsang, Shui-ying Cheng, Cheuk-nam Ling, Cheuk-lam Ho

**Affiliations:** 1 United Christian Hospital, Department of Pathology, Hong Kong, China; 2 CUHK Medical Centre, Department of Pathology, Hong Kong, China

**Keywords:** Carcinoembryonic Antigen, Epidermal Cyst, Pancreas, Spleen

## Abstract

Epidermoid cyst in an intrapancreatic accessory spleen is a rare benign lesion that is difficult to diagnose preoperatively. Cyst fluid analysis for biochemistry markers has been widely used to aid the diagnosis of pancreatic cysts. A high cyst fluid carcinoembryonic antigen (CEA) level (>800 ng/mL) is said to be useful in distinguishing intraductal papillary mucinous neoplasm (IPMN) and mucinous cystic neoplasm (MCN) from other non-mucinous cysts. We herein report a case of epidermoid cyst in an intrapancreatic accessory spleen with abnormally high CEA level (3582 ng/mL) in the cyst fluid, suggesting a potential pitfall in using cyst fluid CEA level as an indicator of mucinous neoplasms.

## INTRODUCTION

Accessory spleen is found in around 15% of the general population. Around 5% of them are located near or in the tail of the pancreas.^1^^,^^2^ Splenic epidermoid cysts are rare and constitute approximately 10% of total splenic cysts.^3^ Epidermoid cyst in an intrapancreatic accessory spleen (ECIAS) is very rare, with only a few dozens of case reports in the English literature to date.^4^^-^43 Although it is a benign lesion, the definitive diagnosis can often be made after excision as an accurate preoperative diagnosis by radiological and endoscopic examinations are difficult.^44^

We herein report a case of ECIAS with abnormally high CEA level in cyst fluid, and a preoperative radiological diagnosis of pancreatic mucinous cystic neoplasm.

## CASE REPORT

**Figure 1:**
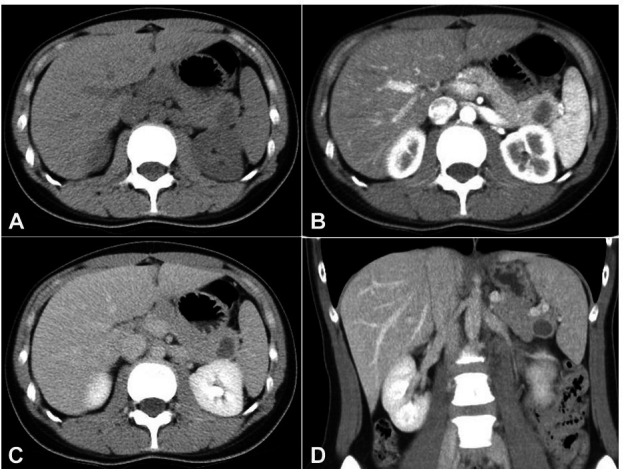
CT scan showed a hypodense and unilocular cyst in the tail of the pancreas, with no internal septation or calcification (**A –** plain, **B –** arterial phase, **C –** delayed phase, **D –** coronal section of delayed phase).

A 28-year-old Chinese woman with an unremarkable past medical history was presented to the hospital with dyspepsia and epigastric pain. She underwent abdominal ultrasound, which revealed a simple cyst at the pancreatic tail measuring 1.4 x1.7 x1.5cm in size, with no internal septa or solid area. A subsequent magnetic resonance imaging revealed a well-defined solitary unilocular cystic lesion in the pancreatic tail region, measuring up to 2.34 cm. There was a small mural nodule in its dependent portion and a minimal enhancement at its thin wall without significant enhancement of the mural nodule. There was no vascular invasion, definite communication with the non-dilated pancreatic duct, or lymphadenopathy. A contrast-enhanced computed tomography (CT) was also performed to further characterize the lesion, which revealed a 1.5 x 1.8 x 1.4 cm mildly lobulated, hypodense, and unilocular cyst in the tail of the pancreas. It showed no internal septation or calcification. The pancreatic duct was not dilated (Figure 1). The radiological differential diagnoses were mucinous cystic neoplasm, cystic form of solid pseudopapillary tumor or mucinous cystadenoma.

**Figure 2:**
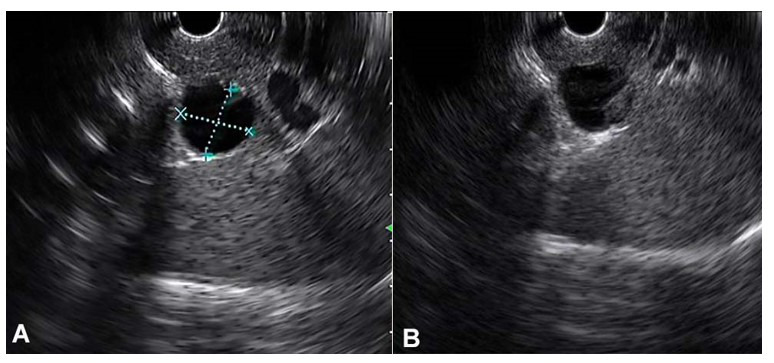
Endoscopic ultrasound with linear echoendoscope revealed a 1.6x1.5cm thinly septated cystic lesion at the pancreatic tail (**A**), with a suspicious 5mm mural nodule (**B**).

Endoscopic ultrasound with linear echoendoscope was also performed and again revealed a 1.6x1.5cm cystic lesion at the pancreatic tail. There was a thin septum within the cyst and a suspicious 5mm mural nodule (Figure 2). The pancreatic duct was not dilated and apparently lacked communication with the cyst. Fine needle aspiration was performed during the examination and yielded a small amount of light brownish fluid. The cyst fluid showed an amylase level of 879 IU/L (>6,800 IU/L suggests pseudocyst instead of mucinous neoplasms,^45^ and < 250 IU/L excludes the possibility of a pseudocyst^46^), and carcinoembryonic antigen (CEA) level of 3582 ng/mL (>800 ng/mL suggests IPMN or MCN^46^). Cytological examination showed no malignant cells.

Based on the radiological findings and cyst fluid biochemistry findings, a laparoscopic distal pancreatectomy with spleen preservation was performed. The surgery was uneventful.

Macroscopic examination of the resected specimen showed a unilocular cyst within the pancreatic parenchyma measuring 1.5 x 1.4 x 1 cm with brownish contents. Microscopic examination showed an intrapancreatic simple unilocular cyst lined by bland -ooking, non-keratinizing squamous epithelium (Figures 33B). Surrounding the cyst was a rim of splenic tissue, featuring typical white and red pulp (Figures 33D and Figure 4). The background pancreatic parenchyma was unremarkable. Immunostains for CD68 and ERG highlighted the littoral cells in the splenic tissue. The squamous epithelium was positive for p63 and CEA (Figure 5). The final diagnosis was ECIAS.

**Figure 3:**
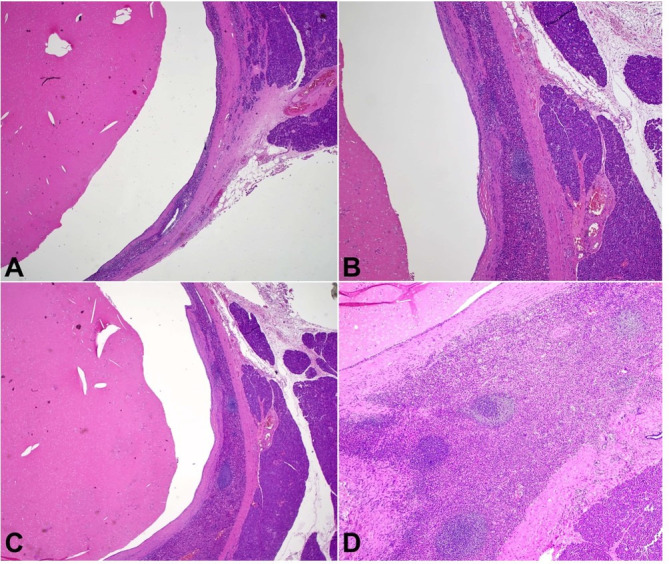
Photomicrographs of the cystic pancreatic lesion. **A** and **B** – H&E sections show a well-circumscribed, intrapancreatic unilocular cyst with a rim of splenic tissue. Normal pancreatic parenchyma is on the right side (20x); **C** and **D** – H&E sections show typical red pulp and white pulp of the splenic tissue (40x).

**Figure 4:**
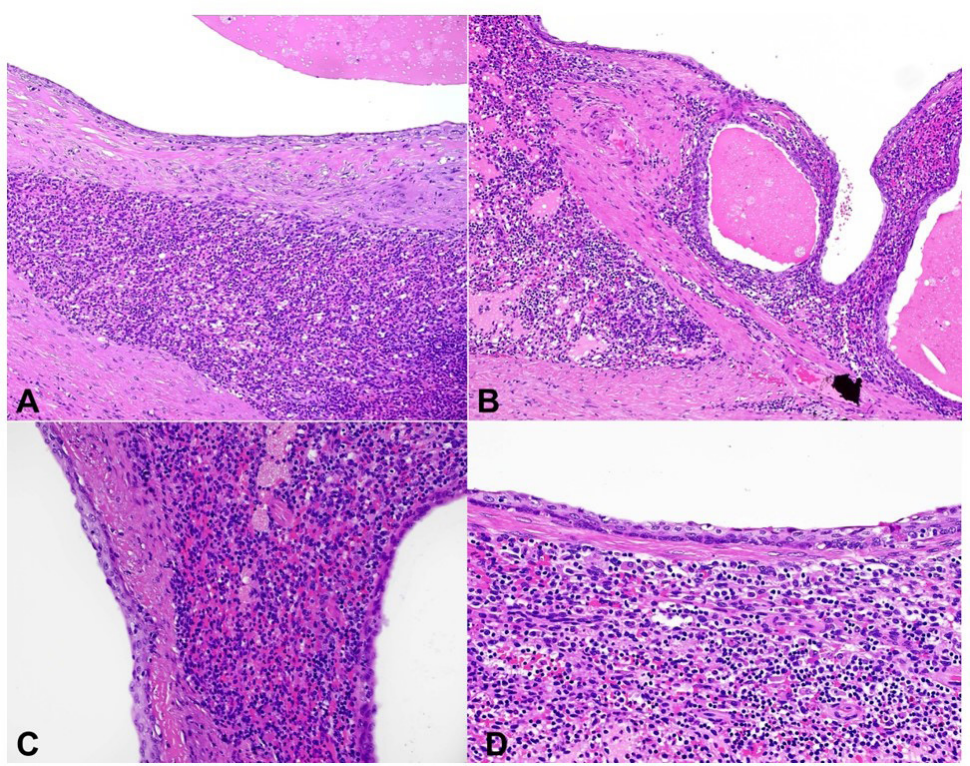
Photomicrographs of the cystic pancreatic lesion. 4A to 4D. H&E sections show cyst lining composed of bland, non-keratinizing squamous epithelium with adjacent splenic tissue (**A** and **B** – 100x; **C** and **D** – 200x).

**Figure 5:**
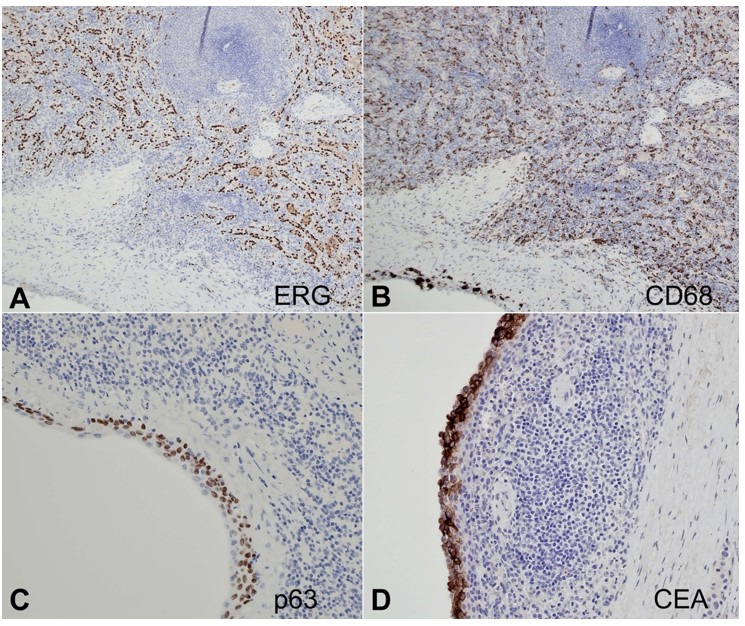
Photomicrographs of the cystic pancreatic lesion. Immunostainings show co-expression of **A** – ERG and **B** – CD68 of the littoral cells in splenic tissue (100x); positivity for (**C**) p63 and (**D**) CEA in the squamous cells (200x).

The patient was discharged one week after surgery, following an uneventful postoperative course.

## DISCUSSION

Accessory spleens are congenital and caused by incomplete fusion of multiple buds of splenic tissue in the dorsal mesogastrium during embryogenesis. The most frequent location is in the splenic hilum and is occasionally found in the pancreas.^1^^,^^2^ The histogenesis of epidermoid cysts is thought to be mesothelial cells included in the spleen parenchyma form an inclusion cyst and then develop squamous metaplasia.^47^ Typical histological findings of an epidermoid cyst of spleen/accessory spleen are benign keratinizing or non- keratinizing squamous epithelial lining within normal splenic tissue.^48^^,^^49^

The accurate diagnosis of ECIAS by preoperative imaging and cyst fluid biochemistry assessment is difficult. Among the reported cases of ECIAS, only five of them have been diagnosed accurately before surgery.

Cyst fluid analysis for cytology and biochemistry markers have been widely used to aid the diagnosis of pancreatic cysts. Cyst fluid carcinoembryonic antigen (CEA) is useful in distinguishing intraductal papillary mucinous neoplasm (IPMN) and mucinous cystic neoplasm (MCN) from other cysts types, with relatively high diagnostic accuracy.^46^^,^^50^^,^^51^ A pooled analysis of 12 studies showed that the cyst fluid CEA cutoff value of >800 ng/mL had a sensitivity of 48% and a specificity of 98% for discriminating IPMN and MCN from other non-mucinous cysts, and very low CEA levels of <5 ng/mL has a very high specificity of 95%, with 50% sensitivity, for non-mucinous cysts, such as serous cystadenomas and pseudocysts.^46^ Cyst fluid amylase is also a useful marker as it is high in pseudocyst, in contrast to intraductal papillary mucinous neoplasm (IPMN) and mucinous cystic neoplasm (MCN).^45^^,^^52^^,^^53^ A cutoff value of 6,800 IU/L for cyst fluid amylase showed a diagnostic accuracy of 69% in differentiating pseudocyst from mucinous neoplasms,^45^ and cyst fluid amylase level of < 250 IU/L had a very high specificity of 98% for excluding a pseudocyst.^46^

Currently, there are no reports regarding cyst fluid biochemistry in ECIAS to date in the English literature. In our case, the cyst fluid showed an amylase level of 879 IU/L, and CEA level of 3582 ng/mL. Although the amylase level was inconclusive, the CEA level was very high and alarming. This high level of CEA and suspicious features in radiological exams prompted the surgeons to offer distal pancreatectomy for the patient. But the final histological diagnosis was ECIAS, suggesting a potential pitfall in using cyst fluid CEA level as an indicator of mucinous neoplasms.

According to a study about CEA level in the epidermoid cyst of the spleen,^9^ the high level of CEA in the cystic fluid are produced by the squamous epithelium lining, and the squamous cells were positive for CEA immunostain. It seems reasonable to apply the findings to ECIAS, and our case is also positive for CEA immunostain.

Histologically, the differential diagnoses of ECIAS include lymphoepithelial cyst, dermoid cyst, and retention cyst. Lymphoepithelial cyst is also lined by squamous epithelium, but there should be abundant lymphocytes in the wall with germinal center formation.^54^^-^56 Dermoid cyst (monodermal teratoma), are composed of sebaceous appendages, hair follicles, and columnar or respiratory epithelium in addition to squamous epithelium.^54^ Squamoid cyst is a distinct type of retention cyst, results from unilocular cystic dilatation of pancreatic ducts due to obstruction. It is lined by squamous epithelium with no lymphocytes in the wall.^57^

## CONCLUSION

ECIAS is a rare benign cystic lesion of the pancreas. It is difficult to diagnose preoperatively and often misinterpreted as other cystic neoplasms of the pancreas, such as IPMN and MCN. Cyst fluid analysis for amylase and CEA have been widely used to aid the diagnosis of pancreatic cysts. A high CEA level is reported to be relatively accurate in discriminating IPMN and MCN from other non-mucinous cysts. However, due to the ability of squamous epithelium in ECIAS to produce CEA, the high CEA level in cyst fluid can be misleading. We should be cautious in interpreting cyst fluid biochemistry results and always correlate with clinical and radiological findings to have a more accurate preoperative diagnosis for pancreatic cysts. The typical histological findings of an epidermoid cyst of the spleen/accessory spleen are benign keratinizing or non- keratinizing squamous epithelial lining within normal splenic tissue. The presence of normal splenic tissue around the cyst lining allows differentiation from other cysts, which are also lined by squamous epithelium, including lymphoepithelial cyst, dermoid cyst, and retention cyst.
